# Better data for better public health decision-making: perspectives on quantifying the intended and unintended impacts of public service modifications

**DOI:** 10.1186/s44263-026-00333-3

**Published:** 2026-10-13

**Authors:** Sangeeta Bhatia, Yehya M. S. Althobaity, Lloyd A. C. Chapman, Josiline Chemutai, Carlo Delfin S. Estadilla, Linda Gansberger, Eva Janoušková, Pauline Nyakairu Karega, Ruth McCabe, Rachel E. Murray-Watson, Tristan M. Naidoo, Mark Pritchard, Bingling She, Timothy B. Hallett, Anna Petherick, Pierre Nouvellet, Anne Cori, Angelique Mavrodaris

**Affiliations:** 1 Medical Research Council Centre for Global Infectious Disease Analysis, Jameel Institute, School of Public Health, Imperial College London, London, UK; 2 Department of Mathematics, Taif University, Taif, Saudi Arabia; 3 School of Mathematical Sciences, Lancaster University, Lancaster, UK; 4 Population Health Sciences, Bristol Medical School, University of Bristol, Bristol, UK; 5 UK Health Security Agency (UKHSA), London, UK; 6 Institute for Global Health, University College London, London, UK; 7 Department of Computer Science, University of Manchester, Manchester, UK; 8 Centre for Tropical Medicine and Global Health, Nuffield Department of Medicine, University of Oxford, Oxford, UK; 9 Blavatnik School of Government, University of Oxford, Oxford, UK; 10 School of Life Sciences, Brighton and Hove, University of Sussex, Brighton and Hove, UK; 11 All Hazards Public Health Response, Health Security Agency, London, UK

**Keywords:** Public health, Public services, Modifications, PHSM, Unintended consequences

## Abstract

Public services such as healthcare, schools, and transport constitute the infrastructure essential for a functioning and productive society. These services may undergo planned modifications because of interventions intended to manage societal challenges, or experience unplanned disruptions resulting from external shocks. For instance, schools might be closed to mitigate the spread of an infectious disease, or when an external shock, such as a heatwave, renders their continued operation infeasible. These modifications to public services, whether planned or unplanned, have both intended and unintended public health outcomes, as well as wider and longer-term societal consequences. To improve our response to various societal challenges, it is essential to assess the relation between the extent of modifications and the intended outcomes, and identify the full spectrum of unintended consequences, both beneficial and adverse. A central pillar of such an exercise would be timely and granular data on the modifications and outcomes - what was done, what happened, to whom, where, and when? However, these data are rarely, if ever, systematically collected. Instead, research into the evaluation of public service modifications relies on sporadic and bespoke data collection. In this perspective, we highlight a critical data gap in tracking public service availability, adaptations, and interventions, illustrating our arguments through two case studies. We examine the challenges in collecting and integrating such data and propose practical solutions to improve data infrastructure and cross-sectoral coordination.

## Background

Public services such as healthcare, education, and transport constitute essential infrastructure for a functioning and productive society. These services may undergo planned modifications because of interventions intended to manage societal challenges, or experience unplanned disruptions arising from external shocks.

A recurring context in which planned service modifications occur is during public health crises, such as the COVID-19 pandemic. A central part of the response to these crises consists of public health and social measures (PHSMs) such as restrictions on gatherings and school and workplace closures that affect the normal functioning of public services. After a myriad of debates about the effectiveness of such measures before, during, and since the pandemic, are we any closer to a consensus? For instance, recognising the impact of school closures, policymakers, researchers, and civil society have often emphasised that schools should be the “last to close, first to open”. However, in a future pandemic, even if age- mediated risk profiles could be rapidly established (itself a considerable challenge), would we know whether to close schools, given the persistent uncertainty surrounding the immediate and longer-term impacts of both illness and school closures? The unfortunate and uncomfortable answer to this question is: no. A primary reason for this negative answer is that we lack robust evidence on the effectiveness of PHSMs deployed in the past, and the existing literature, while substantive, remains fragmented and dominated by low-certainty findings [1–3]. Much of this evidence has been derived from scenario-based modelling analyses, which, while essential, often rely on limited empirical data beyond routinely collected epidemiological indicators. Consider the following: the scientific evidence on the effectiveness of school closures for outbreak control and on long-term societal consequences has long been equivocal [4, 5], particularly so in low- and middle-income countries. Without the systematic collection of data, their continued employment represents a missed opportunity to learn whether, when, and for whom such disruption works, and our blind spot persists.

Public services may also be disrupted by unplanned shocks, such as floods or heatwaves, which can lead to the closure of health facilities, missed clinical appointments, and interruptions to routine services including vaccination [6]. Despite differing fundamentally from planned policy modifications, such disruptions can give rise to substantial downstream health consequences and share many of the same underlying data gaps: service interruptions are rarely recorded systematically, making their health impacts difficult to quantify or compare across events. As with planned service modifications, the lack of data on public service disruptions severely limits our ability to quantify their impact and build a robust evidence base to inform mitigation measures.

**Fig. 1:**
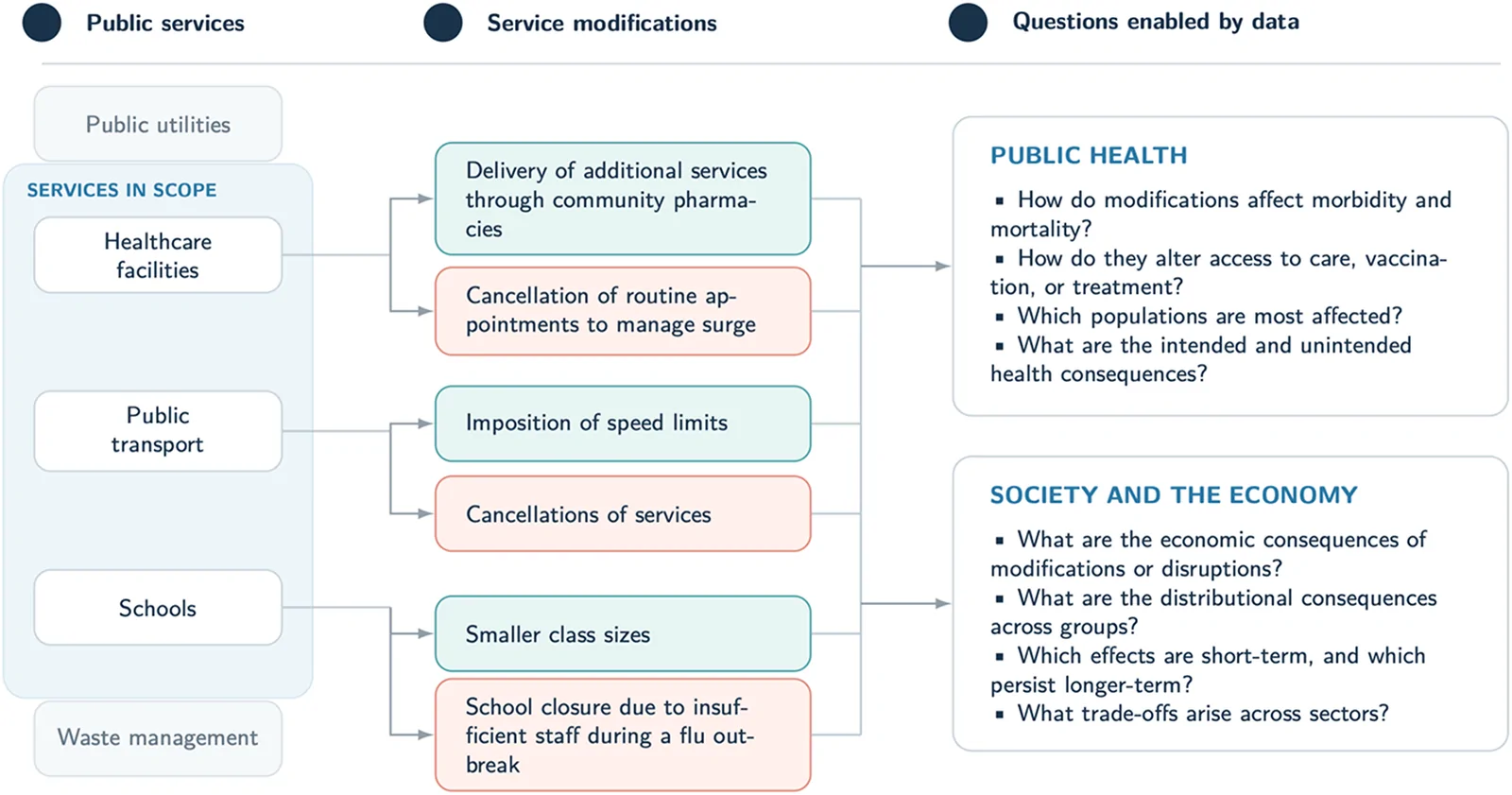
Conceptual framework illustrating the scope and analytical focus of this piece

Here, we argue that systematic data collection on public service modifications, whether planned or unplanned, is essential to quantify their intended and unintended consequences. We recognise that “public services” is a broad and context-dependent concept that varies across countries. Hence, we focus on a subset of services whose modification can plausibly influence population health (Fig. 1). These are (i) healthcare facilities (e.g. primary care, hospital services), (ii) public transport, and (iii) schools. Healthcare services have direct effects on morbidity and mortality, while public transport plays a critical role in enabling access to care [7, 8]. We also include schools as a form of social infrastructure with well-established links to health and wellbeing.

We use “public service modification” to refer to changes in the availability, accessibility, capacity, timing, or mode of service provision arising either from measures introduced to manage a public health threat or from acute external shocks. We first illustrate the multiple pathways through which these services are modified through two case studies - an infectious disease outbreak and a heatwave, both of which have been identified in the United Kingdom’s (UK) National Risk Assessment [9] as events that are likely to occur and have moderate to significant impact. Since the consequences of such alterations tend to overlap, we examine the intended and unintended impacts of these modifications together while recognising that their nature and magnitude will depend on the triggering cause and context. As public health researchers, we place particular emphasis on the health consequences of these modifications, while underscoring their wider and longer-term societal and socioeconomic impacts; we further highlight that data on service modifications can inform a broad range of research and policy questions, with benefits extending well beyond the health sector. To demonstrate this, we draw on examples of policy-relevant analyses enabled by bespoke data collection efforts. We focus primarily on the UK and European contexts, although the gap we highlight is unlikely to be unique to these settings. This focus arises from the authors’ collective expertise and our familiarity with the relevant evidence base. Finally, we discuss key challenges in collecting such data and propose practical recommendations to address them.

## Infectious disease outbreaks

Of the various PHSMs available to policymakers, many will involve the modification of public services as a direct route to reducing opportunities for transmission. Of particular interest are infections that spread through air or droplets or via direct contact, since PHSMs aimed at containing these alter the operation of public services and shared settings, e.g. by closure of sports and entertainment venues, limiting frequency of or density on public transport, implementing national and international travel restrictions [10, 11]. Online education and work-from-home mandates also aim to limit the number of individuals in an enclosed space.

Enhanced susceptibility of children to some respiratory pathogens, practical constraints in sustaining extensive hygiene measures [12], and extensive social mixing in educational and childcare settings [13, 14] make them a key consideration in outbreak control. School closure has been used as a PHSM in previous influenza pandemics and during the COVID-19 pandemic. However, recognising the secondary effects of such measures - ranging from workplace absenteeism caused by increased childcare demands to direct harms on vulnerable children, including food insecurity [15], unreported child abuse [16], and learning loss [17] - other modifications to school services remain part of various pandemic preparedness plans [18, 19]. These include measures such as reducing class sizes, staggered start times or class dismissals [20]. In addition to such system-level modifications, some measures may influence individual access to services e.g. exclusion of children with specific illnesses from educational settings, with significant costs for affected children and their families [21].

Modification of public services could also entail mobilising existing infrastructure to meet new demands. For instance, delivery of vaccines or medical countermeasures may require adaptation of community healthcare facilities or pharmacies. During the 2009 H1N1 pandemic, pharmacies in England served as collection points for antivirals prescribed through the National Pandemic Flu Service [22]. Similarly, schools have routinely been used for targeted vaccination campaigns [23, 24].

## Heatwaves

Heatwaves exert wide-ranging pressures on public services leading to modest to severe disruptions. While these pressures become particularly acute in countries where infrastructures are less resilient, the increased frequency and intensity of heatwaves [25] mean that even regions where high temperatures are common may experience severe disruptions.

Public transport is one of the first services to be affected during heatwaves as authorities take measures to mitigate against the risk of infrastructure failure. For instance, heatwaves in England in 2019, 2022, and most recently in 2026 led to the issuance of “do not travel” warnings, speed restrictions and reduced services for railways [26–30]. On some occasions, flights were suspended due to runway surface defects. Schools and workplaces also experience disruption due to heatwaves. During the 2022 and 2026 heatwaves, many schools in England closed early or modified daily schedules to help pupils and staff avoid the hottest parts of the day [31]. Even in the absence of any measures, heatwaves lead to increased absenteeism as workplaces and schools become uncomfortable and potentially unsafe. Heat-related absenteeism [32, 33] can itself impact service continuity, so that for service users, the operational consequences are effectively indistinguishable from top-down service modification measures.

The increased use of energy-intensive appliances such as air-conditioning units during heatwaves places unprecedented demands on electricity grids, leading to supply failures and outages in some instances [34, 35]. Health services experience increased ambulance and emergency call- outs due to heat-related illnesses such as heat strokes and dehydration. To deal with this increased demand, hospitals have in the past cancelled routine appointments [36].

## Intended and unintended consequences of public service modifications

PHSMs primarily aim to reduce transmission by limiting opportunities for infectious contact. Another strategic objective of these measures could be to preserve healthcare capacity and prevent the collapse of essential services by either stopping transmission altogether, or ensuring that infections are spread over time rather than concentrated in a short period. Indeed, the latter was the stated policy objective of the PHSMs deployed during the early phases of the COVID-19 pandemic in many countries, including the UK [37]. In addition to managing healthcare demand, such measures also buy valuable time to allow for scale-up of pharmaceutical countermeasures (an objective explicitly underscored by the 100-day mission [38]), and strengthening of surveillance, clinical, and laboratory systems.

Many PHSMs are deployed to protect populations at disproportionately high risk such as older adults, clinically vulnerable groups, or those living in congregate settings, by reducing their exposure to circulating pathogens. For instance, school closures are often motivated by children’s enhanced susceptibility to some respiratory infections (relative to adults) and their potential to act as superspreaders [39]. During the pandemic, several major supermarkets in the UK made provisions for prioritised access to grocery delivery slots [40] and offered special shopping hours for elderly and vulnerable customers [41].

Heatwave mitigation measures are implemented with the explicit intention of protecting health, safeguarding critical infrastructure and managing acute pressures on public services. Public service adaptations such as adjusting working hours, particularly for outdoor and manual workers, modifying school schedules, and limiting outdoor activities, are designed to reduce heat exposure and prevent heat-related morbidity and mortality [42]. Similarly, public buildings such as libraries and community centres are repurposed as publicly accessible cooling spaces to provide safe environments for those most at risk, including older adults and energy-poor households. Measures to protect infrastructure, such as imposing speed restrictions on rail services during periods of extreme track temperatures, are intended to prevent structural failure and maintain essential transport services. Within the health system, operational changes such as rescheduling routine appointments or postponing elective surgeries are deployed to mitigate heat-related strain on emergency and acute-care services [43, 44].

Regardless of the underlying reasons, modifications of public services also generate broad unintended consequences that extend beyond the initial hazard. For instance, changes in mobility patterns, whether arising from discretionary behavioural shifts, due to official public health measures, or because of heatwaves or heatwave mitigation measures, have cascading effects on health and social systems. A stark manifestation of these long-lasting consequences of short-term changes is the reduction in uptake of routine healthcare due to changes in the demand and supply of health services. During the COVID-19 pandemic, surveys consistently showed widespread avoidance of routine healthcare, with over a third of adults in some settings reporting missed check-ups or chronic disease reviews due to movement restrictions or fear of infection [45–47]. Similarly, emerging evidence also shows increased rates of missed appointments during heatwaves [48, 49]. Conversely, the reallocation of healthcare capacity to deal with additional stress due to outbreaks or heatwaves, has in the past led to cancellation or postponement of non-urgent services. In England, for example, the delivery of routine diabetes care processes fell sharply in the first year of the pandemic, contributing to excess non-COVID-19 mortality among people with chronic conditions [50]. Across both contexts, these reductions in contact between patients and healthcare providers result in poorer [51] health outcomes that persist even when the underlying causes change.

Similarly, whether triggered by policy decisions or environmental shocks, the closure of schools sets off a cascade of effects that touch learning, labour, short- and long-term health [52], and social mobility. Emerging evidence establishes a link between school closures during the pandemic and reduced physical activity and health [53]. An extensive (and growing) body of literature supports the link between unscheduled closure of schools, even when for a short duration, and measurable loss in learning outcomes. For instance, a 21- week closure (the average duration across 41 countries during the COVID-19 pandemic) is estimated to translate into the loss of almost an entire year’s worth of learning [54, 55]. This learning loss is projected to lead to loss of lifetime earnings and poorer long-term health outcomes, illustrating that these impacts are not concentrated in time. Crucially, these losses percolate along existing socioeconomic fault lines, disproportionately affecting pupils already in most deprived communities, thereby entrenching current inequalities. Beyond the impact on pupils, school closures also cause productivity loss through parental absenteeism [56, 57], especially in health and care sectors where women comprise a large proportion of the workforce.

## Robust data needed to quantify consequences

The existing evidence on the downstream consequences of public service modifications is derived largely from ad-hoc datasets assembled for the purposes of individual studies, and is therefore fragmented and sparse. Collecting these data is a resource- and time-intensive process, and is, by definition, not systematic - thereby resulting in potentially incomplete or inconsistent coverage across places and periods.

In 2010, lacking uniform reporting requirements, the Department for Education in England estimated snow-related disruption from local-authority and media websites [58]. Closures are now captured through pupil-attendance codes, but published data do not provide an event-level record of individual school closures. Researchers examining the educational effects of COVID-19-related closures in Sicily also constructed a bespoke dataset by coding individual regional administrative acts, as no harmonised dataset of school-closure days was available [59]. Similarly, researchers in the United States have assembled a list of school closure days by emailing school representatives for historical records to address questions such as the effect of snow-related closure on students’ test scores [60, 61], while similar datasets have been separately assembled to investigate the link between school closures and workplace absence. In contrast, in isolated instances where data have been collected systematically [62, 63] (notably during the COVID-19 pandemic), they have demonstrated substantial value underpinning a wide range of analyses from tracking population behavioural changes before policy announcements [57] to the effect of school closures on parental labour supply [64].

The COVID-19 pandemic also prompted a proliferation of expert-led initiatives (such as ACAPS [65], Covid-19 pandemic policy monitor (COV-PM) [66], and Response2covid19 [67]) that collected data on a range of PHSMs. These initiatives developed detailed codebooks and applied consistent coding of government measures across countries. Some of them incorporated semi-automated extraction from official announcements or media sources, but all depended primarily on sustained manual curation. Volunteer-driven efforts, most notably the Oxford COVID-19 Government Response Tracker (OxCGRT [68]) and CoronaNet [69], mobilised large coding teams to curate detailed data on PHSMs. These efforts have enabled robust comparative evaluations of policy timing, intervention effectiveness [70] and health-system responses across jurisdictions. Crucially, once established, the datasets rapidly generated value well beyond their original remit: researchers in economics and governance [71], education, mobility [72], and environmental science [73] repurposed the same structured data to investigate firm resilience, learning losses, travel-behaviour changes, democratic accountability and even short-term air-quality improvements. This cross-domain uptake illustrates the wider potential of timely, comparable data on service modifications to support policy analysis in areas far removed from infectious-disease control.

## Challenges in collecting public services modification data

The various pandemic-era initiatives demonstrate that prospective and rapid collection of data on public services modification is feasible and highly valuable. Nevertheless, the winding down of these efforts after the acute phase of the crisis also points to systemic logistical and operational hurdles in sustained data collection. Here we catalogue some of the challenges to illustrate the complexity of the task.

### Public service modification data are highly heterogeneous

The first challenge lies in defining public service modification, which implicitly contains the notion of a baseline or “normal” service delivery. In many settings, public service provision is patchy and inconsistent [74, 75], making it difficult to measure specific modifications arising from policy choices or external shocks. In such contexts, an alternative approach is to move away from attempting to directly classify discrete modifications and instead focus on the systematic collection of longitudinal data on public service provision. This allows both baseline conditions and deviations from them to be inferred from observed patterns over time, rather than specified a priori.

Even where public service modifications are well-defined and episodic, they vary widely in scale, specificity, and the level of detail with which they are reported. Some disruptions are highly localised - for example, the closure of a single school [76], the suspension of a specific clinic’s services [77] - while others are broad measures [78], such as citywide lockdowns or national transport restrictions. This variability reflects the severity of the challenge and the operational realities but it poses a foundational challenge for any system attempting to capture such events in a structured and comparable way. Highly structured and standardised formats could improve comparability and enable automated analysis but at the same time, risk stripping away crucial context - such as whether a service was partially reduced or fully suspended, or whether a modification applied universally or only to specific subpopulations. Conversely, unstructured free-text entries preserve contextual detail but limit the ability to synthesise data across settings. One way to reconcile this trade-off is to adopt tiered, modular data collection frameworks, in which data are captured at multiple, nested levels of granularity. This allows both coarse and highly detailed observations to coexist and be integrated within a single coherent structure, rather than requiring uniform levels of detail across all settings.

Public service modifications are often reported with imprecise or delayed information about timing, location, and scope [79]. Uncertainties in the start and end dates of an intervention, the exact boundaries of affected areas, or the specific services impacted can propagate into downstream analyses and lead to divergent interpretations of the same event. We argue that such uncertainties are in fact a core feature that should be explicitly represented in data collection systems. This can be achieved, for example, by recording time intervals (akin to uncertainty intervals) rather than fixed dates to indicate the start and end of a modification, approximate or fuzzy geographic boundaries, and the extent of or confidence in reported impacts. This, in turn, enables downstream analyses to account for uncertainty directly, rather than relying on implicit assumptions about precision.

### Public service modification data are highly scattered and continually evolving

Beyond the challenge of defining what to collect, data collection is further complicated by the heterogeneous and sometimes ephemeral ways in which public service modifications are communicated. Existing policy trackers have had to assemble information from multiple sources, including government websites and statements, legal documents, news reports, and social media. Modifications communicated through channels that tend not to be archived may therefore be more difficult to retrieve retrospectively.

Further, public service modifications may be announced, revised, reversed, or clarified over time, and these changes must be captured accurately to avoid misleading records. This compounds the difficulties in establishing robust processes for data verification and quality assessment.

This fragmentation across sources and over time can be addressed through data systems that aggregate information from diverse channels, track provenance, and maintain versioned records. Emerging AI-based methods such as large language models can be leveraged to monitor diverse information sources, including local news, community announcements, and government statements across multiple languages.

In addition to formal reporting mechanisms, participatory approaches such as data donation via smartphones or reporting by frontline workers and service users offer a means of capturing locally observed changes that may not be systematically documented through official channels. Data could also be “donated” by private sector organisations that operate or support public services, such as transport providers, as these actors often hold detailed, real-time information on service availability and usage. Precedents for such approaches already exist, such as the “Data for Development” [73] initiative in which the telecom provider Orange shared anonymised call records, as well as the widespread release of mobility indicators by technology companies such as Google and Apple during the COVID-19 pandemic.

Humanitarian open mapping initiatives such as https://www.missingmaps.org/ and Wikipedia are powerful examples of sustained crowd-sourced data collection supported by layered verification processes and mechanisms to manage noise, duplication, and misinformation. The practices underpinning Wikipedia - transparent edit histories, consensus-based moderation, tiered user privileges, machine-assisted vandalism detection, and a culture of documentation - demonstrate that open, crowd-based knowledge systems can be robust and self-sustaining when supported by clear governance norms.

## A reusable data ecosystem

We have argued that a systematic registry of public service modifications could provide the missing data layer needed to investigate a wide range of policy questions. What might a data ecosystem with such a registry at its core look like? Recent World Health Organisation [19] guidance calling for the monitoring of PHSM policies through a harmonised framework and minimum set of critical data elements provides a useful starting point. We propose extending this approach to capture changes in service provision resulting from PHSMs or external shocks.

The called-for registry would contain, for each modification of interest such as a school closure, a record of the modification - for example, whether the school was fully or partially closed, or introduced a new provision - where and when the change occurred, why it occurred, and how long it lasted. Each record would also hold information on the extent of modification, populations affected, baseline provision (where possible), data provenance, indicators of data quality and completeness, and any uncertainty in the recorded information. Crucially, this infrastructure need not be built from ground up. Alterations in service provision are already captured (even if partly) through various surveillance streams (e.g. pupil attendance data with school closure codes, public transport timetables and cancellations among others) but these vary in coverage, resolution, and accessibility. Common metadata and identifiers, together with a unified discovery and access layer, could make these distributed sources more findable, accessible, interoperable and reusable [80]. Coupled with emerging PHSM evaluation frameworks and pre-specified protocols designed for rapid activation during emergencies [19, 81], such a registry could provide the implementation data needed to generate timely evidence on effectiveness and unintended consequences.

**Fig. 2:**
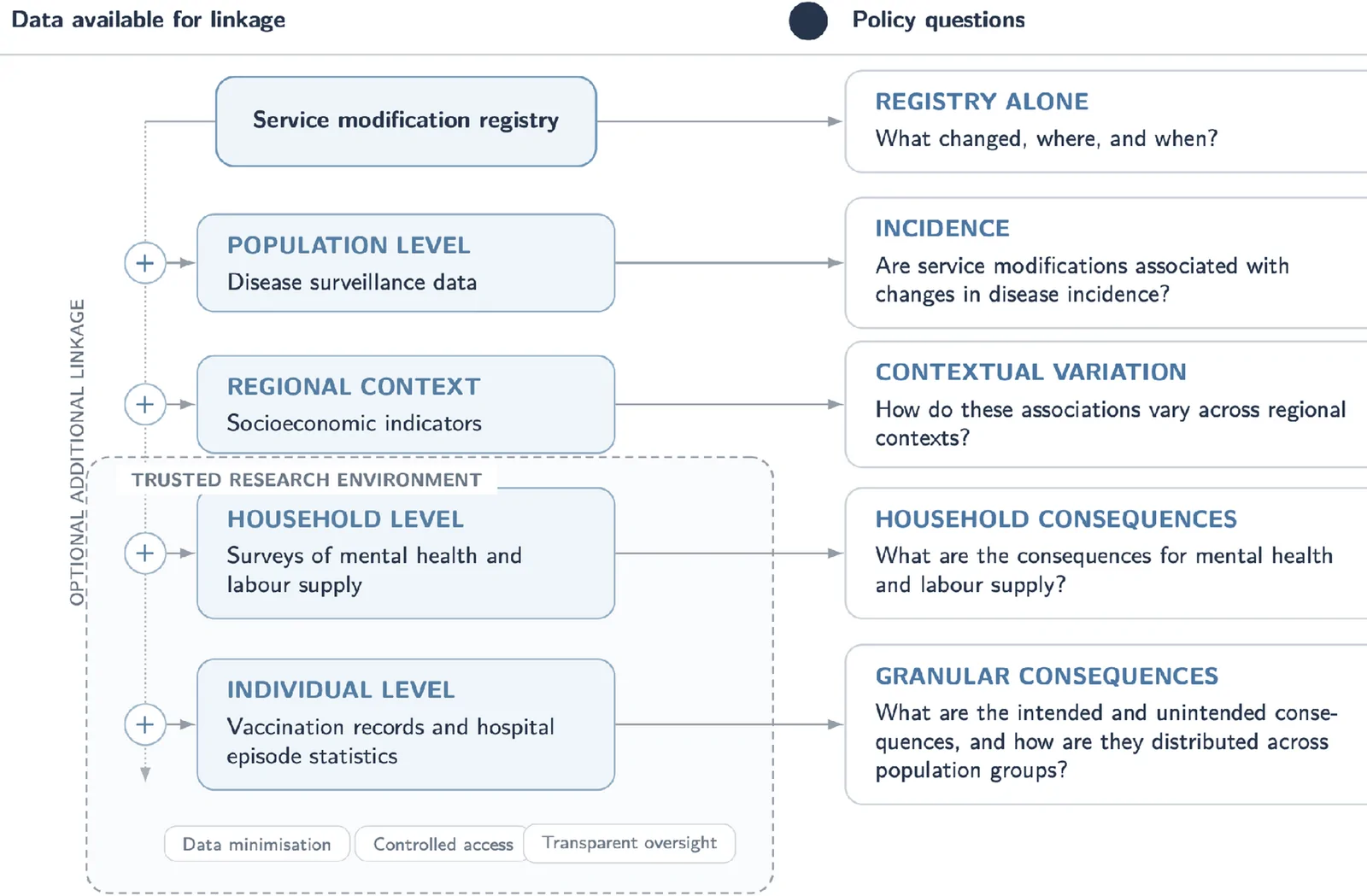
Illustrative blueprint for a data ecosystem with public service modification data at its core. Linkage with routinely collected data sets enables research into policy-relevant questions, with examples given here for an infectious disease outbreak

A systematic registry could on its own support descriptive monitoring and comparisons of service availability. Its analytical potential would emerge through linkage with other datasets that describe the context in which service modifications occurred and their consequences (Fig. 2). For instance, linking service modification data with population-level disease surveillance data allows researchers to explore associations between modifications and changes in disease incidence [64, 82]. Additional regional socioeconomic indicators can further reveal how these associations vary across contexts. Linkage with household surveys can generate insights into such varied outcomes as mental health and labour supply [83]. Finally, linkage with individual-level records and outcomes, such as vaccination records [84] and hospital episode statistics [85], can enable granular estimation of the intended and unintended consequences of these modifications and how these consequences are distributed across population groups. The combination of data needed would depend on the question being addressed, allowing the ecosystem to develop and generate value incrementally. Established data-linkage models such as Trusted Research Environments coupled with data minimisation, and transparent oversight can facilitate useful research while protecting privacy and addressing concerns about access and governance.

Establishing such an infrastructure would raise important questions concerning ownership, maintenance, sustainable funding, privacy, and access. Existing research infrastructures provide several models: centrally stewardship (e.g. UK Biobank [86]), coordinated access across distributed datasets (e.g. CLOSER [87]), federated analysis of sensitive data (OpenSAFELY [88]and DARWIN EU [89]. Small-scale pilot initiatives could first demonstrate the value of a public services modification registry, providing a basis for funders and relevant public bodies to invest in an appropriate long-term governance model.

## Conclusions

Public services constitute the core of our shared civic lives. Changes to these services have long-lasting consequences that reverberate through time and space. Data on public service modifications are essential for understanding both the intended and unintended consequences of such modifications, yet they remain among the least systematically collected forms of evidence in public health and disaster management. When such data have been assembled - whether during pandemics, extreme weather events, or local service disruptions - they have repeatedly enabled insights across epidemiology, economics, education, and infrastructure policy.

The myriad challenges in prospectively collecting data on public service modifications mirror long-standing challenges in multiple disciplines, and the wide variety of approaches adopted provides valuable insights into how future data collection systems could be designed, governed, and maintained. Disaster losses information management systems such as DesInventar [90]and the recent efforts by the United Nations Office for Disaster Risk Reduction, United Nations Development Programme, and World Meteorological Organization to develop enhanced disaster tracking systems [91] serve as important parallels as well as points of connection for public service modifications data.

These challenges, and potential solutions to them, are also likely to vary between high- and low- and middle-income countries. Given our focus on the UK and Europe, we have not examined the commonalities and contextual differences - this is an important avenue for future research.

The absence of sustained, prospective data systems therefore represents a critical and preventable gap. Inspired by the Coalition for Epidemic Preparedness Innovations’ (CEPI) “prepare, transform, connect” framework for accelerating pharmaceutical countermeasures, we propose an analogous three-pronged strategy to address this unmet need. We must **prepare** by developing the foundational protocols, governance structures, and technical standards necessary for real-time data collection during crises. We should **transform** the response to future pandemics and disruptions by building robust analytical pipelines and embedding meaningful feedback loops between data, modelling, and decision-making. To do this, we must **connect** stakeholders across academia, public agencies, industry partners, media organisations, civil society, and affected communities to ensure that such systems are co-designed, equitably governed, and operational before emergencies arise. Investing in these capabilities now will strengthen preparedness for the next outbreak or extreme event.

## Data Availability

No datasets were generated or analysed during the current study.
